# Management of a Nursing Unit in a Temporary COVID-19 Specialized Hospital in Wuhan, China

**DOI:** 10.1017/dmp.2020.373

**Published:** 2020-10-12

**Authors:** Chunmei Luo

**Affiliations:** Department of Orthopedics, Xinqiao Hospital, Army Medical University, China; Second Department of Infectious Diseases, Taikang Tongji COVID-19 Specialized Hospital, Wuhan, China

**Keywords:** COVID-19, infection control, infectious disease, nursing management

## Abstract

The purpose of this article was to summarize the experience of conversion and management of a nursing unit in a newly revised coronavirus disease 2019 (COVID-19) specialized hospital during the outbreak of COVID-19 in Wuhan, China. Six characteristics of management were included: nurse selection and training, transformation of ward layout, nurse position setting, quality control, humanistic care, and safety and comfort of individual protection. Orderly and efficient nursing management during COVID-19 treatment is very important to ensure the quality of clinical nursing, improve the cure rate and avoid the infection of nurses. This practical experience of the establishment and management of the nursing unit can provide reference for the nursing management of other public health events, such as the treatment of infectious diseases.

In December 2019, multiple cases of pneumonia with unknown cause, which were caused by a novel coronavirus,^1^ emerged in Wuhan, China. The International Classification Committee of the Virus designated the virus as severe acute respiratory syndrome coronavirus 2 (SARS-CoV-2); and the World Health Organization (WHO) named the disease coronavirus disease 2019 (COVID-19).^2^ To stop the epidemic in Wuhan, the National Health Commission sent medical teams from all over the country to Wuhan beginning January 24, 2020.

On February 13, 2020, our medical team was sent to Wuhan Taikang Tongji Hospital, which was a general hospital under construction. It was temporarily transformed into a COVID-19 specialized hospital, with 1016 beds in 19 departments and 1400 staff that came from 55 hospitals. The author served as a chief nurse in 1 infectious disease department that had 60 beds. From February 19 to March 30, a total of 127 confirmed COVID-19 cases, including 45 severe cases and 9 critical cases, were admitted to our department. Except for 3 patients, who were transferred to the intensive care unit (ICU), the cure rate was 97.6%. Two patients fell down and 2 patients encountered a first-degree burn without medical consequences. No patient died in the department; we called it “zero patient deaths.”

Every nurse was skillful at wearing and taking off PPE. No mistakes were made in wearing or taking off PPE in a green or red zone. Negative nucleic acid test was found twice on staffs’ hands, tables, a door handle, masks, computers, and in the air in the green zone. Headache and dizziness were found in 71.84% of nurses in the red zone, and nausea was found in 40.78% of nurses. No vomiting or fainting occurred. One nurse encountered a sterile needle puncture in the red zone without medical consequence. Sleep disorders were reported, and pills were needed in 16% of nurses. One nurse caught a cold, with a temperature of 37.4°C for 2 d. No one asked for leave during the whole period. Throat swabs tested negative twice for staff when the hospital was shut down—we called it “zero staff infection.” To provide a reference for front-line nursing management in the treatment of COVID-19, we summarized the experiences of management, including nurse selection and training, transformation of ward layout, position setting, quality control, humanistic care, and safety and comfort of personal protection.

## NURSING MANAGEMENT

### Nurse Selection and Trainings

Because COVID-19 is a severe, highly infectious respiratory disease, each team needs 3-level protective suits to work as not only nursing but also house keeper in the red zone for at least 4 hours, and takes high risk of infection. Therefore, the nurses, on a volunteer basis, came from respiratory, emergency, cardiovascular, gastroenterology, neurology, and other specialized departments, and priority was given to those who had good physical and psychological health without related medical history and experience with epidemic infectious disease care, wearing and taking off protective clothing skillfully, and more than 2 years’ experience with respiratory intensive care.^3,4^ Among the 50 nursing staff of our group, 1 had experienced severe acute respiratory syndrome (SARS) in Xiaotangshan Hospital, 2 had experienced Ebola virus disease in Liberia, 14 had experienced an earthquake disaster medical rescue and medical exercise, 20 had experience with intensive care and ventilator use, and 3 had experienced extracorporeal membrane oxygenation (ECMO) nursing training. For example, as the chief nurse, the author encountered nursing experiences with SARS and Ebola infectious diseases. The group included 33 nurse practitioners, 16 nurses in charge, and 1 associate professor of nursing. The ratio of males to females was 13:37. The average age was 31.92 years (± 3.94 years) old.

The special trainings were as follows: COVID-19 prevention and treatment seminar, guidelines for the diagnosis and treatment of COVID-19, ventilator use, disinfection and isolation technology for COVID-19 and self-protection technology for medical staff, including the wearing and taking off of personal protective equipment (PPE). Seven key points of high-risk nursing procedures were practiced, such as nursing skills on noninvasive and invasive ventilator nursing, tracheotomy nursing, sputum aspiration nursing, atomization inhalation, nasopharyngeal swab sampling, vessel puncture techniques, endotracheal intubation, and fibrobronchoscope operation, and teaching, practice, and individual testing on wearing and taking off PPE (Table 1).

**TABLE 1:** Wearing and Taking Off PPE

Wearing	Taking off	
	Undressing Room1	Undressing Room 2
1. Hand hygiene	1. Hand hygiene	1. Hand hygiene
2. N95 mask	2. Disposable apron	2. Inner pair of boot covers
3. Head cover	3. Hand hygiene	3. Hand hygiene
4. Goggles	4. Face shield and head cover	4. Protective gown
5. Gloves 1	5. Hand hygiene	5. Hand hygiene
6. Protective gown	6. Surgical mask	6. Second pair of gloves
7. Gloves 2	7. Hand hygiene	7. Hand hygiene
8. Boot covers 1	8. Outer pair of boot covers	8. Goggles
9. Disposable apron	9. Hand hygiene	9. Hand hygiene
10. Head cover	10. Outer pair of gloves	10. Inner head cover
11. Surgical mask	11. Hand hygiene	11. Hand hygiene
12. Face shield		12. Inner pair of gloves
13. Gloves 3		13. Hand hygiene
14. Boot covers 2		14. N95 mask
15. Signature		15. Soak and disinfect soles
16. Perform hand hygiene		16. Hand hygiene

The special PPE practices were to ensure proper covering of skin, air tightness of protective equipment, and no over tightening or intolerance. Hydrocolloid dressing, foam dressing, wound paste, etc. were used to prevent facial pressure injury caused by mask nose clip, goggles, and other protective equipment. Before donning PPE, baby diapers were worn and (or) energy and water were consumed according to personal preference to reduce the factors that may lead to intolerance. To ensure that everyone can wear and take off PPE properly, infection control monitoring was set up to supervise the process of nurses’ wearing and taking off PPE through video monitoring.

### Conversion of Ward Layout

**FIGURE 1:**
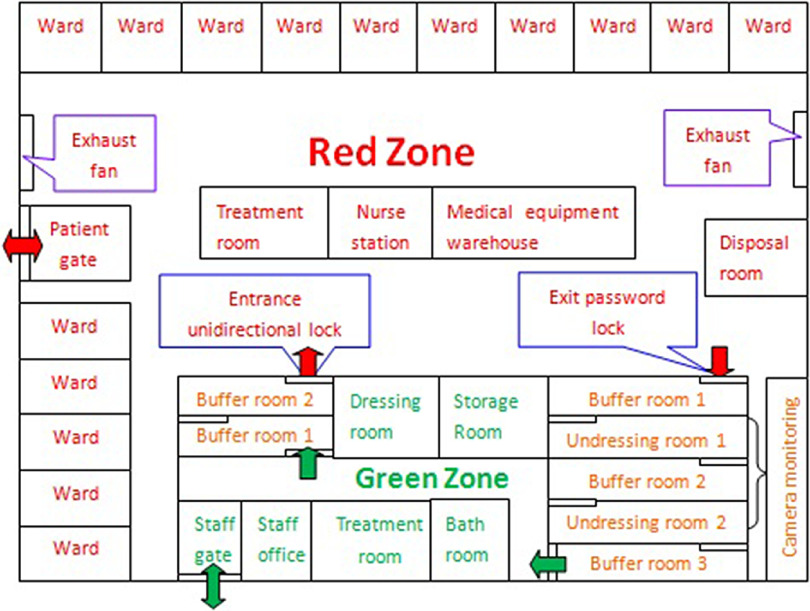
Revised Layout of the Department.

Because the Wuhan Taikang Tongji Hospital was a general hospital without an infectious disease department, the layout of the ward area was revised to meet the requirements of an infectious disease ward. The ward was divided into 3 areas: the contaminated zone (red zone), the relatively clean zone (green zone), and a buffer zone between the red and green zones. Two buffer rooms from green zone to red zone were unidirectionally locked. A password lock was set at the exit from the red zone to the green zone only for medical staff. The green zone could only be reached through 3 buffer rooms and 2 undressing rooms. All door joints were fitted with sealing strips to ensure air tightness. Because it was a nonnegative pressure isolation ward, exhaust fans were specially installed at both ends of the corridor in the ward. The mechanical exhaust fans continuously operated for 24 h/d to form negative-pressure ventilation. The ceiling of the ward and the working area was a movable cover plate, which was connected to the air. The ceiling was closed with plastic film to prevent air from the red zone flowing into the green zone. The whole ward was closed by management. Patient access only opened when patients entered and left the hospital or went out for examination, accompanied by medical staff. The camera monitoring system was installed in the ICU and the undressing room to monitor the condition and trend of critical patients and whether the process of PPE removal was compliant (Figure 1).

### Nurses Position Setting

The nursing staff were divided into a nursing management group and a clinical nursing group. The members of the nursing management team were all chief nurses with management experience. They worked for 8 h/d but no more than 4 h in the red zone. The position setting of the nursing management group was as follows: infection control supervisor, for the supervision of the entire process of dressing and undressing as well as the supervision and inspection of the implementation of various infection control measures; equipment manager, for the requisition, sorting, and in/out registration of all equipment, consumables, protective articles, and materials; office nurse, for handling and checking the medical orders, entering the body temperature list, handling in and out of the hospital, oral medicine dispensing, and management of medicine in and out of warehouse; quality control nurse, for the nursing procedures, inspection of nursing documents, night rounds, and critical illness rescue (Table 2).

**TABLE 2:** Position Setting and Work Time

Crews	Mon	Tue	Wed	Thu	Fri	Sat	Sun
Group 1 (6)	0-4	4-8	/	8-12	12-16	16-20	20-24
Group 2 (6)	4-8	/	8-12	12-16	16-20	20-24	0-4
Group 3 (6)	/	8-12	12-16	16-20	20-24	0-4	4-8
Group 4 (6)	8-12	12-16	16-20	20-24	0-4	4-8	/
Group 5 (6)	12-16	16-20	20-24	0-4	4-8	/	8-12
Group 6 (6)	16-20	20-24	0-4	4-8	/	8-12	12-16
Group 7(6)	20-24	0-4	4-8	/	8-12	12-16	16-20
Office nurse(2)	8-18	8-18	8-18	8-18	8-18	8-18	8-18
Quality control nurse(2)	14-21	8-18	14-21	8-18	14-21	8-18	14-21
Equipment manager(2)	8-18	8-18	8-18	8-18	8-18	8-18	8-18
Infection control supervisor	8-18	8-18	8-18	8-18	8-18	8-18	8-18
Chief nurse	8-18	8-18	8-18	8-18	8-18	8-18	8-18

Each clinical nursing group included 6 members in a fixed arrangement, according to gender, specialty, seniority, work experience, critical respiratory disease nursing experience, etc. At least 2 persons in each group could operate the ventilator skillfully, and each group included 1-2 male nurses. The clinical nursing group entailed 4-h shifts in the red zone, so there were 6 groups covering 24 h/d. The posts of the clinical nursing group were set as follows: the team leader, who was responsible for the management and coordination of the whole shift group; the duty nurse, for management of the treatment, condition observation, health education, personal care, basic care, psychological care, accompanying nurse for inpatient transfer, and in and out of hospital transportation of patients, etc. The nurses with severe disease nursing experience were put in charge of critical patients. Each group had an infection control liaison person who was responsible for the implementation of infection control measures, and each group included a materials administrator who was responsible for the sorting, homing, registration, warning, and supplementary filling of materials (Table 2). Several special nursing teams were set up according to their specialty, including a team for treating wounds, thrombus, using the ventilator and providing airway care, providing diabetes care, and a team responsible for peripherally inserted central catheter (PICC).

The workflow for each position and each group was formulated according to the workload of each group. Full preparations were made in the green zone, and only doctor’s orders for immediate execution were performed in the red zone. In the green zone, the doctor’s order was processed; the doctor’s order form and execution of standing orders forms were printed; the oral medicine was checked; the injection medicine was prepared; the blood sampling inspection barcode was pasted, etc. In the red zone, only printing of the temporary execution form, delivery of oral medicine, injection, blood sampling, and other operations were performed. WeChat (a live social contact app) group was established for multiple mobile phones in the red and green zones, which was convenient for information transmission between red and green zones and connection process management and high efficiency operation between red and green zones.

### Quality Control

**FIGURE 2:**
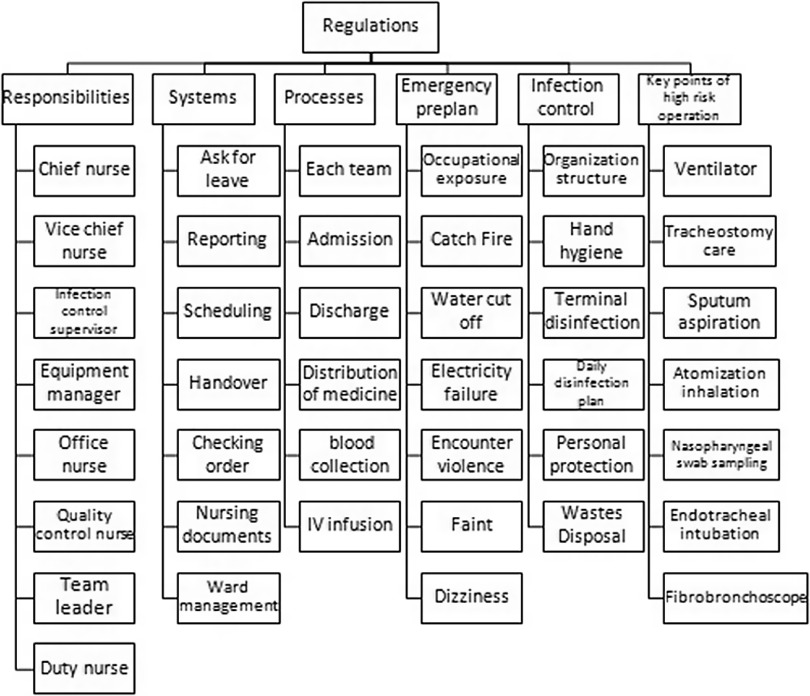
Quality Control Regulations.

The nursing team preliminarily established 8 items of responsibilities, 7 work systems, 6 nursing work processes, 6 infection control systems, 7 key points of high-risk nursing operations, and 7 emergency plans (Figure 2). Once problems arose in the ward, the chief nurse provided feedback in the WeChat group every day and held quality control meeting 1-2 times accordingly. Nurse meetings for all nurses, except those on duty, were held once a week regularly. Video or audio conferences were presented if the staff could not be present. The team leader in charge checked the nursing quality and documents every day, sorted out the documents by stages, preliminary drafted the documents at the peak stage of large-scale admission, and focused on the quality of critical disease care records at the stage of concentration of critical patients. During the stage of sorting out the discharged medical records, the doctors and nurses coordinated with each other one-on-one to sort out the medical records, strictly regulating the management of high-risk drugs, anesthetic drugs, first-aid drugs, and equipment in the ward to ensure quality and safety.

### Humanistic Care

It has been reported that 96.2% of COVID-19 patients have significant posttraumatic stress symptoms, and half of health-care workers (49.8%) considered psycho-educational services to be helpful.^5^ Feelings of isolation, fear of family infection, and fear of neurosis were the main factors leading to anxiety and depression. The nursing group advocated the concept of people-oriented nursing, which entailed establishing a sincere and kind caring relationship between nurses and patients for solving the practical problems of patients. A WeChat group for doctor-patient communications could be used to unblock the communication channels between patients and their families, reply to the questions of patients and their families in a timely manner within the group, appease patients’ emotions, and often let patients and their families make video calls to obtain family support.^6^ Wish trees and cartoons for encouraging patients were arranged in the corridor of the ward to create a warm and positive atmosphere and help patients build up their confidence in disease recovery. Baduanjin mindfulness exercises psychology in traditional Chinese Medicine were integrated into the nursing of patients with COVID-19. Psychological experts provided group counselling online and individual counselling for patients with prominent psychological problems. Nurses who had received psychological training provided face-to-face counselling and reduced the anxiety, depression, insomnia, and other problems of patients.

### Safety and Comfort of PPE

Donning and doffing of PPE was practiced and individually tested by infection control specialist beforehand (Table 1). Attention was paid to checking the air tightness of protective equipment and whether it covered all skin in addition to testing its comfort to avoid adverse reactions and intolerance caused by over-tightening. We used hydrocolloid dressing, foam dressing, and wound paste to prevent facial pressure injury caused by mask nose clip, goggles, and other protective equipment. Before putting on PPE, diapers were worn and/or energy and water were consumed on an individual basis to reduce factors that could increase intolerance. To ensure that everyone could wear and take off PPE without any problems, infection control monitoring was set up to supervise the process of nurses’ wearing and taking off PPE through video monitoring.

In the 3-level protection, nurses could encounter vomiting, fainting, needle stabbing, and other accidents in the red zone. An emergency plan was set up in advance. In the early stages, work time in the red zone was initially capped at 2-3 h so that the nursing staff could gradually adapt to it. In the case of headache, dizziness, or other discomfort, staff were encouraged to sit down immediately and rest as necessary. If these adjustments did not relieve the discomfort, 2 staff were to withdraw from the red zone at the same time. The red zone was set up with an emergency room, which was equipped with face screens, gloves, boot covers, alcohol, and other items in the case of an accident, such as exposure.

## DISCUSSION

### Motivation and Care Are at the Core of Nursing Team Construction

In this anti-epidemic mission, nurses have had a very heavy workload, bearing various roles such as nurse, cleaner, porter, etc. Nurses are at high risk of infection because of their close contact with patients. Accordingly, 50.4% of medical staff working at the front line of COVID-19 have depression symptoms, 44.6% have anxiety symptoms, and 34.0% have insomnia symptoms.^7^ In Italy, nurses working at the front line of COVID-19 have even committed suicide.^8^ As a nursing manager, it is necessary not only to motivate nurses to do their best but also to take care of the physical and mental health of nurses.^9^ We adopted the group model to arrange group shifts and delineated clear responsibilities. Therefore, our team worked with high efficiency and mobilized the initiative of the nurses. To ensure the physical and mental health of nurses, we allocated human resources according to the workload and addressed the sub-health status and psychological problems, such as dizziness, weakness, insomnia, anxiety, or depression.

### Sufficient Preparation Is the Guarantee of Zero Infection of Staff

It is very important to prepare for training, ward layout, medical equipment, and so on. Otherwise, the staff may be exposed to the risk of infection. Because most nurses have no prior experience of epidemic infectious disease care, they should be re-trained in a standardized way, especially with regard to wearing and taking off of PPE. The layout, access routes, and procedures must fit in with the strict isolation standards of the infection ward, with 1-way access and no air convection between the red area and the green area. Therefore, we staggered the position of the doors and placed sealing tape at the door edges to ensure air tightness. We put up obvious zone signs and PPE process signs as reminders to the medical staff. In addition, we prepared the PPE and sterilizing items that would be needed in each area in advance, and we ensured that these things were put in place. We checked the water pipes, lights, drainage, and other conditions of the whole ward one by one to ensure that all problems were solved before any patients were admitted. We also prepared mobile phones and walkie talkies in the red area and green area for cross-area communication. We installed video surveillance in some wards and undressing rooms to monitor the condition of serious patients and supervise the undressing process.

### Integrating Cross-Zone Processes Can Improve Efficiency

Because the hospital was transformed from a general hospital into an infectious disease hospital, every department was divided into red and green zones, with no semi-contaminated zone (yellow zone) and transfer window. Moreover, elderly patients with underlying diseases and serious illness could account for the majority of the COVID-19 patients. As much of the workload as possible should be accomplished in the green in advance, to avoid the workload of nurses in the red zone becoming very heavy. Therefore, we set up treatment rooms and nurse stations in both the red and green zones. In the green area, we dealt with orders, printed implementation sheets, dispensed drugs, pasted barcode labels, and other preparatory work, and then we accomplished the direct implementation of nursing work after entering the red zone. Thus, integrating cross-zone processes can reduce the workload of nurses in the red area and improve work efficiency.^10^

### Nursing of Complication and Humanistic Care Are Indispensable

Elderly persons may account for the majority of the COVID-19 patients. Some of them also have diabetes, cerebral infarction, Alzheimer’s disease, Parkinson’s disease, and other diseases.^11,12^ Other problems are also encountered, such as multiple pressure injuries on the skin, incontinence of stool and urine, poor self-care ability, etc., which may lead to various risks, such as asphyxia, aspiration, falling, thrombus, pressure injury, malnutrition, etc., bringing great challenges to nursing work. Thus, nurses need to correctly evaluate the management risks and ensure the continuous implementation of basic nursing measures. Moreover, patients with COVID-19 often have anxiety, fear, and other emotions related to worrying about their outcome and isolation from their relatives, etc. These patients need more humanistic care from nurses to strengthen their confidence in overcoming the disease. H. Catton. RN, chief executive officer of the International Council of Nurses, also praised the sense of responsibility and love shown by Chinese nurses during this dangerous epidemic.^13^

### Safety and Comfort of Wearing PPE Should be Considered

COVID-19 is highly infectious and is classified as a class B infectious disease but is managed as a class A infectious disease. On February 21, 2020, the Chinese Centers for Disease Control and Prevention announced that 1716 medical personnel in China had been infected with COVID-19.^14^ Therefore, we used 3-level protection in medical treatment. At the beginning of this work, everyone paid special attention to the strictness of protection and thought that the best state of protection was to make it as tight as possible. Unfortunately, headaches, dizziness, nausea, and other discomforts frequently occurred when working in the red zone. We immediately examined 2 people who had been assigned to the emergency exit of the red zone and analyzed the cause of their discomfort, finding that it may have been related to the over-tightening of safety goggles and face screens. Then, we directed our attention toward adjusting the tightness of goggles and screens, which reduced the discomfort greatly. We suggest that it is necessary to pay attention to comfort and tolerance at the same time as tight protection.

## CONCLUSIONS

The practice of nursing during COVID-19 is urgent and full of hardship and danger. The nurses are under great pressure both physically and mentally. Our experiences in the establishment and management of the nursing unit in the infection ward of a COVID-19 special hospital provided the following insights: The selection and training of nurses formed the basis for carrying out the work. Revising the ward layout was conducted under the premise of infection control. Setting reasonable positions and process management made the work orderly and efficient. Quality control was necessary to ensure work quality and reduce risk. Humanistic care made patients feel relaxed and built up their confidence to overcome the disease. In addition, we should pay more attention to the tolerance of protection and the sub-health and psychological problems of nurses to ensure their physical and mental health. This experience can be a reference for the establishment and management of nursing units in response to sudden outbreaks of infectious diseases.
